# Metrics of student dissatisfaction and disagreement: longitudinal explorations of a national survey instrument

**DOI:** 10.1007/s10734-023-01004-0

**Published:** 2023-03-29

**Authors:** A. Mark Langan, W. Edwin Harris

**Affiliations:** 1 Department of Natural Sciences, Manchester Metropolitan University, Chester Street, Manchester, UK; 2 Agriculture and Environment, Harper Adams University, Newport, UK

**Keywords:** Student surveys, Dissatisfaction, Disagreement, Neutrality, Satisfaction

## Abstract

This study explores dissatisfaction and neutrality metrics from 12 years of a national-level undergraduate student survey. The notion of dissatisfaction is much less prevalent in the narratives surrounding student survey outcomes, and the underpinning metrics are seldom considered. This is despite an increasingly vociferous debate about ‘value for money’ of higher education and the positioning of students as consumers in a marketised sector. We used machine learning methods to explore over 2.7 million national survey outcomes from 154 institutions to describe year-on-year stability in the survey items that best predicted dissatisfaction and neutrality, together with their similarity to known metric predictors of satisfaction. The widely publicised annual increases in student ‘satisfaction’ are shown to be the result of complex reductions in the proportions of disagreement and neutrality across different survey dimensions. Due to the widespread use of survey metrics in university league tables, we create an anonymised, illustrative table to demonstrate how UK institutional rankings would have differed if dissatisfaction metrics had been the preferred focus for reporting. We conclude by debating the tensions of balancing the provision of valuable information about dissatisfaction, with perpetuating negative impacts that derive from this important subset of the survey population.

## Background

A key component of the marketisation of higher education (HE) has been rising tuition fees and the notion that university students are customers or consumers of education (Hazelkorn, 2015). Despite opposition to neoliberalism in the sector (Budd, 2017; Baird & Elliott, 2018), the increasing reliance on metrics to drive policy and practices is indisputable to the extent that it has been described as ‘metric fetishizisation’ (Spence, 2019) and ‘datafication’ of HE (Williamson et al., 2020). A significant contributor of the shift towards capturing and quantifying educational parameters has been the acquisition, reporting, and usage of the views of undergraduate students (Adisa et al., 2022). Surveying university student ratings of their educational experiences befits the contested ideology of them as consumers evaluating the ‘service’ or educational ‘product’ that they have experienced (Naidoo, 2018). There are benefits for universities being associated with desirable metric outcomes, particularly the enhancement of national and international reputations as a consequence of favourable, publicly available information (Locke, 2014). In the UK, this effect is galvanised by the incorporation of metrics into governmental evaluations of institutional quality and publicly available third-party schema, such as league tables and ‘good university’ guides (Gunn, 2018; Locke, 2011).

Hazelkorn (2015) highlights the heightened influence of metrics worldwide being driven by their inclusion in league tables. The practice intensifies competition between institutions in a reputation race and may lead to contestable data-driven decision-making by senior managers. Thiel (2019) provides evidence of deleterious impacts of the effects on the academic community of management practices resulting from responses to survey outcomes, drawing on notions of power and governmentality (following Foucault, 1977). There is significant evidence of negative effects of metricisation in many organisational sectors (Muller, 2018). Baird and Elliott (2018) articulate the problem with performance metrics underlying issues with performativity, whereby academic staff try to improve metrics without believing in their utility. Spence (2019; p.771) builds on this by highlighting the narrow focus that educational metrics provide and how this leads to “the illusion that [universities] are managing and controlling something more effectively, but doing so produces consequences that run counter to the spirit of the overall exercise”. On the other hand, there is evidence that university surveys have played a role in enhancing (learning) experiences through empowerment of university students and their student unions, as institutions respond to published outcomes (e.g. Brown, 2011; Robinson & Sykes, 2014). It has also been argued that self-reported educational survey metrics can act as proxy measures of the success of the pedagogical approaches used (Barefoot et al., 2016), and there is a well-established assertion that tertiary level learners can make valid judgements of the quality of their educational experiences (Ramsden, 1991). However, there are complex, interacting factors that influence survey participants and many counter views (Langan, 2022). This emphasises the need for research into the nature of information about university courses that should be used to inform prospective students about their quality and potential value (Gunn, 2018).

In those countries that they are used, self-reported surveys can augment a culture of HE institutions focusing and responding to overly simplistic quantified ratings (Sabri, 2013). There is a particular emphasis in the literature of evidence and viewpoints from the UK and Australia for tertiary-level education, as their national survey instruments derived from a common ancestor (Yorke, 2015). These countries were early adopters of large scale, self-responding surveys of HE student views. We refer to Adisa et al., (2022, 2015) and Hazelkorn (2015) for wider insights into the proliferation and worldwide use of this type of survey instrument, often being linked to notions of satisfaction with university experiences.

‘Satisfaction’ remains a contested term, and surveys of student views normally provide a highly constrained and a highly simplified view of the complex notion of satisfaction (Elliott & Shin, 2002). Despite the limitations, purported measures of satisfaction are widely reported in research literature, government outputs, and general media. In contrast, the reporting of dissatisfaction and disagreement is much less prevalent in narratives about higher education metric outputs. When there is an option of neutrality in survey instruments, levels of disagreement do not simply represent an inverse of agreement meriting separate explorations of all three outcomes (agreement, neutrality, and disagreement; see Smithson et al., 2015). It seems a particularly valuable opportunity to explore disagreement metrics in an era of vociferous debate about ‘value for money’ for university students and a need to avoid disappointment and complaints from HE participants (Khan & Hemsley-Brown, 2021).

There is a long history of evidence that satisfaction and dissatisfaction represent different ‘dimensions’, providing different information about views rather than merely being simple opposites of each other (Herzberg et al., 1959; Cadotte & Turgeon, 1988). Explorations of customer dissatisfaction are widespread, most commonly in research literature for marketing and business-related disciplines (e.g. Iyer & Muncy, 2008). However, this is now highly developed in other sectors, including a range of sophisticated measures of service quality in HE, such as the adaptations of SERVQUAL (e.g. Pham et al., 2022). Personal (student) expectations are known to play key roles in governing subsequent dissatisfaction with the lived reality and, subsequently, complaint behaviours. For example, notions of unfulfilled expectations that lead to customer complaints include defective products, poor service quality, and unfulfilled promises (Nimako, 2012). Dissatisfaction with products and services is, generally, the precursor to complaints (Huefner & Hunt, 2000) and can result in the loss of consumer demand through negative brand association (Juric et al., 2015).

In the current study of the UK’s National Student Survey (NSS), we necessarily use a widely applied and simple interpretation of the notions of student satisfaction and dissatisfaction, derived directly from the metric responses to the survey item that enquired ‘Overall, I am satisfied with the course’. Participant satisfaction is therefore represented by metric outcomes of Likert responses 4 and 5, and dissatisfaction by 1 and 2 to this survey item. This simplistic, metrics-driven approach is established in literature that explores the UK’s survey instrument (e.g. Bell, 2022). The neutral response, the mid-value of 3, is interpreted as neither satisfied nor dissatisfied. In a broader sense, we follow the ethos of Oliver (1989; also cited in Elliott & Shin, 2002) who interpreted student satisfaction as the “favourability of a student’s subjective evaluation of the various outcomes and experiences associated with education”. Our reliance on metrics means that we make no attempt to develop theoretical frameworks around notions of (dis)satisfaction. Further context about these concepts is provided by Douglas et al (2008) and Khan and Hemsley-Brown (2021).

There is substantial literature that identifies complex and interacting factors that shape university student satisfaction, varying in the level of influence academics can manage them (Langan, 2022). Examples include graduate employment (Lenton, 2015), campus and social life (Elliott & Shin, 2002; Douglas et al., 2015), attainment (Nicholson et al., 2013), personality traits (Braun & Zolfagharian, 2016; Dean & Gibbs, 2015), and ‘lecturer’ attitudes and behaviours (Farr-Wharton et al., 2018). It is argued that delivery of experiences that heighten student satisfaction requires employees of a university to adhere to the principles of quality customer service (Douglas et al., 2015). The so-called service encounters as students engage with university systems can lead to ‘moments of truth’ as experienced by customers, and these form an important part of their overall impression of the whole service (Carlzon, 1989; Pham et al., 2022). Perceptions of such experiences are largely driven by interactions between people; however, other aspects (such as institutional reputation, physical environment, senses of professionalism and helpfulness, and personal convenience) may significantly influence perceptions of general service quality (Galloway, 1998). Senses of belonging in the educational journey and loyalty to the course or institution can further modify holistic perceptions of the wider student experience and influence student survey ratings (Devinder & Datta, 2003; Braun & Zolfagharian, 2016).

The contentious student-as-customer perspective is a well-known viewpoint that modifies narratives of the student experience in complex ways (Budd, 2017; Gremler & McCollough, 2002; Iyer & Muncy, 2008). Lala and Priluck (2011) contrast UK student experiences with general customer service frameworks, highlighting how comparatively simple it is to switch between commercial service providers compared to the complexity of leaving a UK university course. In HE systems such as those of the UK, university students are subject to high ‘switching costs’, not just financially but also due to the disruption of the educational process, difficulty in finding and applying to a new course, and emotional challenges of leaving one place and adapting to new physical and social spaces (Singh, 1990). This complexity may make students more inclined to complain than to exit, and this discontent is captured at least, in part, by survey comments and metrics (Langan et al., 2017). Student decision-making varies across the world as a consequence of the influences of many interrelated educational, cultural, and societal factors such as student expectations, course experiences, and ultimate learner outcomes (Khan & Hemsley-Brown, 2021).

Drivers of university-level student dissatisfaction have been unearthed through qualitative explorations of student views. For example, Douglas et al. (2008) used critical incident analysis to elucidate the drivers of satisfaction and dissatisfaction using questionnaires and hand-written narratives in Douglas et al. (2015). This body of work provides valuable evidence of the importance of themes of ‘communication’ and ‘attentiveness’ as key contributors to satisfying and dissatisfying aspects of undergraduate student experiences. These themes have been related to metric predictors of student satisfaction (Langan & Harris, 2019), and there is consensus that very few survey items strongly predict the overall satisfaction metric in the UK’s National Student Survey (NSS) instrument (Burgess et al., 2018; Langan & Harris, 2019; Langan et al., 2013; Parapadakis, 2020). These relate to ratings of courses running smoothly, perceived effectiveness of teaching, and, to a lesser extent, the support they receive. There remains minimal research connecting the accompanying text comments to the output metrics. Langan et al. (2017) found that a higher proportion of negative (compared to positive) text comments about aspects of course organisation and teaching was associated with lower overall satisfaction. These were also the aspects of the student experience commented upon most in the participant text responses. There remains a paucity of evidence that dissatisfaction/ disagreement metrics are explicitly made available in HE worldwide, although the notion may be raised in local reporting (pers. obs.). Dissatisfaction metrics are reported in other areas, such as medicine (e.g. Xu et al., 2017), architecture (e.g. Pin et al., 2018), digital resource use (e.g. Tilahun & Fritz, 2015) as well as broader consumer views (e.g. Gamboa et al., 2015).

We focus on the UK’s National Student Survey (NSS) that was launched in 2005. The self-responding instrument was introduced as a development of the Australian Course Experience Questionnaire (CEQ; see Ramsden, 1991) and intended to provide publicly available data to inform prospective students about where to study before being used for quality assurance and quality enhancement purposes (Richardson, 2013). For more information about its history, refer to Richardson et al. (2007), and Kandiko Howson and Matos (2021). The original instrument harvested views from over two and a half million university student participants and reported mostly steady, year-on-year increases in its metric outputs during its lifetime from 2005/6 to 2016/17 (Burgess et al., 2018). This was despite indications of ‘levelling off’ of some dimensions such as teaching before the instrument was modified (Langan & Harris, 2019). The rise in metric outcomes was widely interpreted as the product of changes implemented by UK institutions as a consequence of responding to student views (Burgess et al., 2018).

After 10 years of usage, the NSS instrument underwent a review (see Callender et al., 2014). The original core set of questions was later modified and relaunched in the 2017/18 academic cycle, providing 12 years of data from the original instrument (Office for Students, 2022). The new survey instrument included additional dimensions and reworded items but retained much of the original UK national survey instrument reported on here, which comprised the six dimensions: teaching; assessment and feedback; academic support; organisation and management; learning resources; and personal development (see Table 1). Much of the original survey wording remained unchanged in the new version, including the holistic rating of overall satisfaction “Overall I am satisfied with my course” (Q22 in the original instrument, becoming Q27 in the new instrument). This single item is often considered to be a survey dimension on its own (Cheng & Marsh, 2010; Fielding et al., 2010).

**Table 1:**
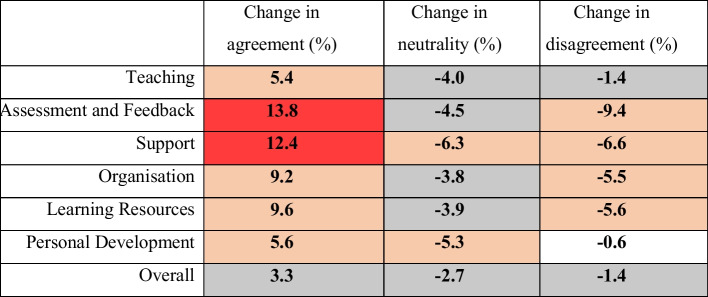
Overall changes in median dimensional levels of agreement (ratings of 4 and 5), neutrality (ratings of 3), and disagreement (scores of 1 and 2) combined from 2005/6 to 2016/17. Background indication: red = greater than 10% change; orange = greater than 5% change; grey = greater than 1% change; white = less than 1% change

It is now indisputable that the UK’s national survey instrument has had significant impacts on the UK’s HE sector, emanating from reporting of student satisfaction and agreement metrics, which are incorporated into a governmental-level Teaching Excellence Framework or ‘TEF’ (a simplistic grading of institutional quality; Bell, 2022). There remains dynamism and debate about the use of NSS metrics, including the halving of their contribution to the TEF (https://wonkhe.com/blogs/the-office-for-students-has-designed-a-new-tef/). The UK HE ecosystem is viewed as susceptible to tensions associated with metrics (Hazelkorn, 2015), and we suggest that international audiences can benefit from our dissection of the lifetime of a national survey instrument as means to unveil metric drivers associated with student satisfaction and dissatisfaction.

The current study provides new insights into national student surveys by reporting longitudinal patterns of disagreement and neutral metrics, in contrast to discourses dominated by narratives about student success and satisfaction. Machine learning is used to rank the importance of predictors of student dissatisfaction. Metric outcomes are also tabulated for illustrative purposes as a way to demonstrate how institutional metric rankings change as a result of the emphasis being moved from agreement to disagreement. The research aims are to use the original survey dataset to (1) document long-term trends in metrics of agreement, disagreement, and neutrality; (2) identify the important metric predictors of agreement, disagreement, and neutral outcomes; and (3) explore the impact of a shift of metric focus from student agreement to disagreement.

## Methods

The quantitative approach used follows Langan and Harris (2019) who explored levels of student agreement across institutions in the UK NSS. The survey is administered by an independent agency to acquire information about the views of undergraduate students in their third (often final) year of study. The output metrics are intended to inform the choices of stakeholders such as potential future students, acting as a surrogate measure of the quality of the courses (Surridge, 2008). Participants provide ratings of agreement for positively worded statements about university experiences (1–5; strong disagreement to strong agreement). For consistency with previous research (e.g. Langan et al., 2013), we refer to survey items using the notation ‘Q’ (even though the survey items are not formulated as questions), e.g. ‘Q1’ for survey item 1. For each item, there is the option of neutrality (a rating of 3). Therefore, expression of disagreement (ratings of 1 or 2) is not necessarily an inverse of agreement (ratings of 4 or 5).

Only the final core survey item in the UK survey enquires directly about respondent satisfaction with their course. The term ‘satisfaction’ in this context is also commonly used in the wider literature to refer to other survey dimensions as well, such as students being considered to be ‘satisfied’ with teaching in reference to items in the teaching dimension. Strictly, this usage is not representative of the wording of these survey items, which are ratings of the experience and not an explicit expression of respondent satisfaction (see Langan & Harris, 2019). Here, we use terminology such as ‘agreement’ and ‘disagreement’ with the positively worded survey item statements. The terms ‘satisfaction’ and ‘dissatisfaction’ are used in reference to the outcomes of the survey item enquiring about overall satisfaction with the course.

The current study utilises the full lifetime of the original UK survey instrument to provide a longitudinal view of the metric outcomes, drawing on 12 years of returns to explore consistency and patterns over time. The revised instrument in 2017 was very similar in general terms but added new survey dimensions, including learning opportunities, learning communities, and the student voice (Pollet & Shepherd, 2022). The core components of the original survey instrument were retained, often with identical or very similar wording (Callender et al., 2014).

### Data extraction and analysis

Nationally available data for 12 years of the UK NSS were extracted from 2005 to 2016 (i.e. for the academic years from 2005/6 to 2016/17) for all subjects and institutions (http://www.hefce.ac.uk/learning/nss/data/). We selected only institutions with more than 500 respondents during each year for analysis and used raw (non-benchmarked) data for all student typologies. This resulted in a set of 154 institutions and 2,741,654 returns across the 12 years. Random forest analysis (Breiman, 2001) was used to interrogate the dataset to rank individual survey items Q1–Q21 in their association with classification of overall dissatisfaction, neutrality, or satisfaction for survey item Q22. This approach is widely used in classification analysis, where the task is to identify associations between a set of predictor variables (here, individual survey items Q1 to Q21) and a categorical dependent variable (here, the three possible outcomes of Q22). In general terms, random forest analysis is a powerful supervised machine learning algorithm that creates multiple decision trees, creating a “forest” of outcomes, each slightly different. We used the metric survey items to classify the outcomes of the overall satisfaction question (agree, neutral, or disagree). To create the different trees, the analysis samples subsets of the overall dataset, selecting a subset of variables and cases (i.e. randomly choosing certain respondents and survey items to generate a unique outcome, or tree). The outcomes were aggregated for an overall ranking of which survey items best predicted the satisfied, dissatisfied, or neutral responses.

For our analysis we selected five predictors per tree and 500 trees, using a selection of 70% of rows for each tree (Liaw & Wiener, 2002). The relative importance of each predictor in the model structure was assessed as an equally weighted mean percentage of increase in mean squared error (MSE) and its Gini score (the reduction in accuracy of models excluding the target variable), ranking survey items in order of their importance in predicting dissatisfaction (Genuer et al., 2010). We qualitatively compared the importance of different variables in predicting each of the overall satisfaction class. Finally, a simple university ranking table was created based on overall satisfaction and compared it to an alternative ranking based on dissatisfaction to facilitate examination of differences between these approaches. All analyses were conducted using the statistical package (R Core Team, 2022).

## Findings

### Trends of survey dimensions

**Fig. 1:**
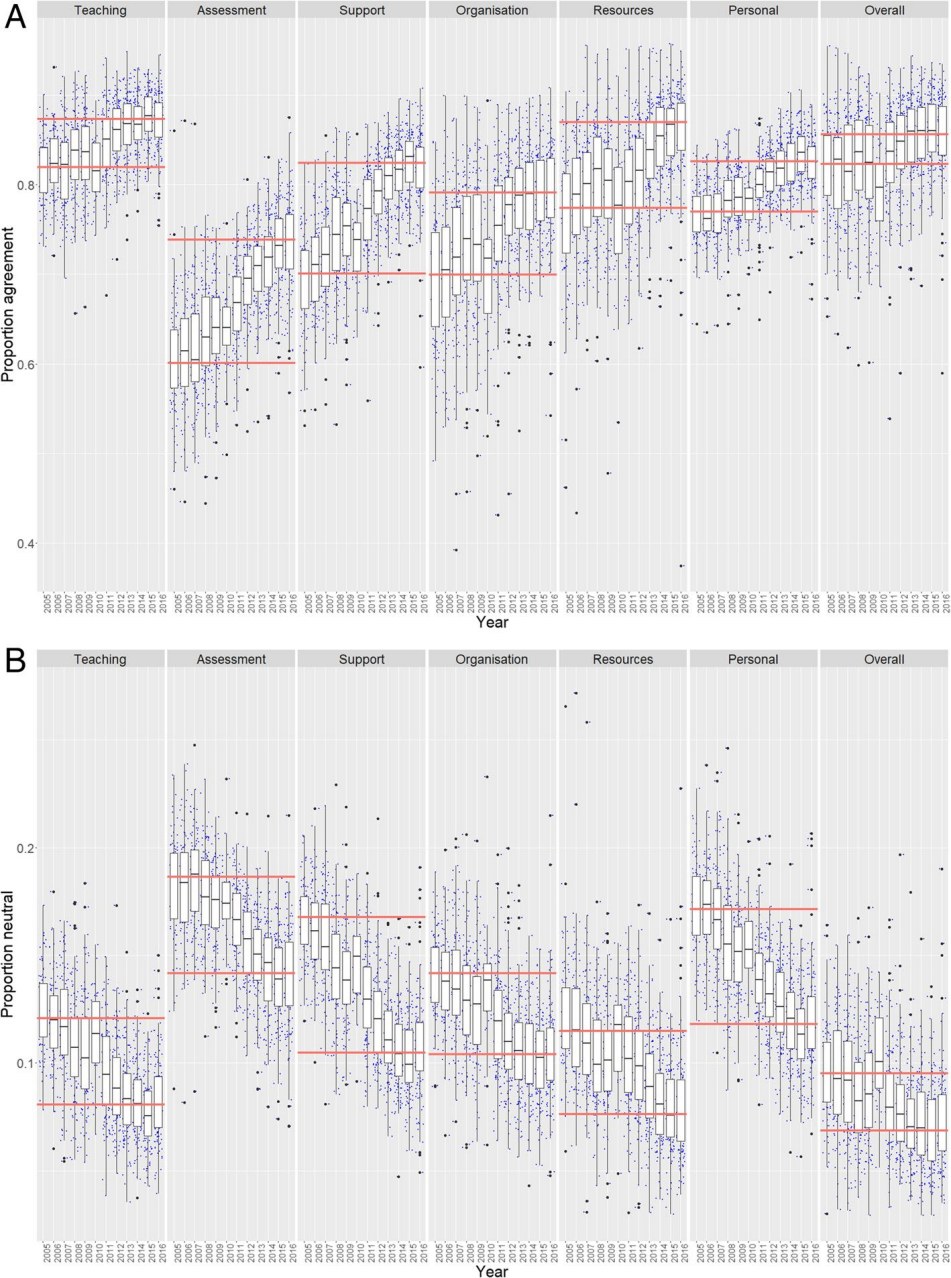

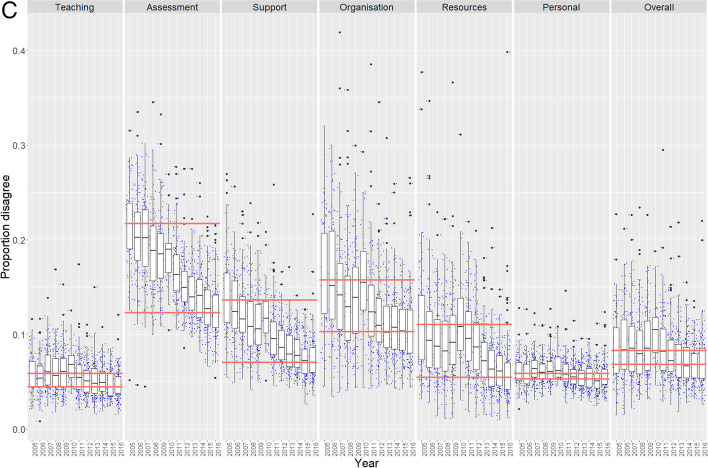
Longitudinal trends in **A** agreement (Q22 ratings of 4 and 5), **B** neutrality (rating of 3), and **C** disagreement (ratings of 1 and 2). Responses for each of the 12 years are shown for all survey ‘dimensions’, which average multiple items for survey instrument dimensions (e.g. ‘Teaching’ is represented by four survey items Q1–Q4). Boxplots represent the inter-quartile interval, and whiskers indicate the 90% confidence interval. Horizontal red lines indicate median ratings for each dimension at the start (2005) and the end (2016) of the survey instrument lifetime

Longitudinal trends of agreement, disagreement, and neutrality are shown in Fig. 1, highlighting the gains in ratings of 4 and 5 responses over the original survey’s lifetime. Over time, students became more ‘satisfied’ and less ‘dissatisfied’ together with a diminishing proportion of neutrals. Generally, there were very few upper outliers within the agreement metrics (i.e. few institutions significantly over-performed in the sense that they were associated with extremely high levels of satisfaction), and, also, there were few lower outliers with disagreement metrics (i.e. few institutions significantly over-performed in the sense that they had significantly lower levels of dissatisfaction). However, outliers representing institutions in the extremes of the datasets indicate that certain dimensions, particularly those of Organisation & Management and Learning Resources, had examples of extreme levels of under- or over-performance in the sector.

There were only minimal levels of disagreement at the start of the survey for dimensions such as teaching and personal development. Thus, year-on-year changes were relatively minor, and levels remained around 5% of the sample population. Most dimensions were relatively stable in their minimal levels of disagreement by the end of the survey instrument’s lifetime, arguably with the exceptions of assessment and feedback and learning resources that still appeared to be declining. In contrast, there were clear declines in the levels of neutrality across all dimensions and at comparatively similar magnitudes.

The variation between the survey dimensions in the reductions of neutrality and disagreement that ultimately drove the increases in agreement is summarised in Table 1. In terms of absolute values, reductions in neutral responses to bolster agreement, rather than this being a consequence of reductions in disagreement, were most pronounced for the dimensions of personal development (5.3% out of 5.6% increase) and teaching (4.0% out of 5.4% increase). Increases in metric outcomes of the other dimensions were more balanced in terms of the relative contributions to agreement of reduced levels of neutrality and disagreement. This was with the exception of assessment, which exhibited the highest level of enhancement overall, and, in this case, the gains were drawn mostly from reductions in levels of disagreement (9.4% out of 13.8%).

### Predictors of dissatisfaction and neutrality

**Fig. 2:**
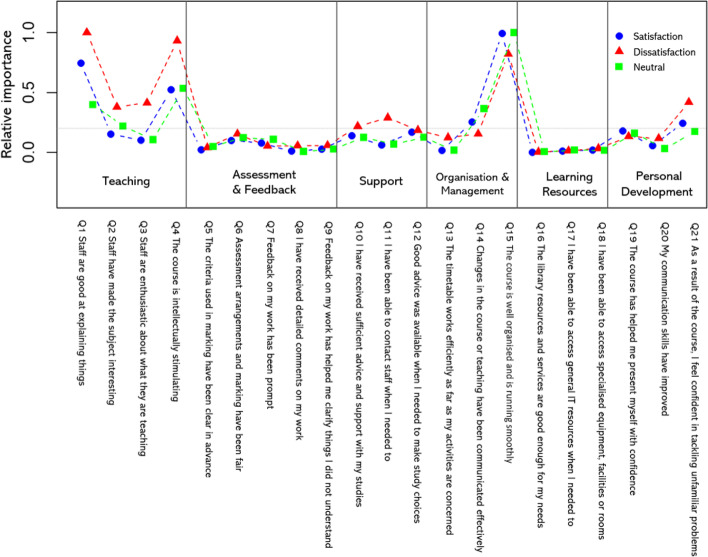
Trends in the predictive power of the NSS survey items (Q1–Q21) based on their relative importance as predictors of Q22. Patterns are shown for predictors of satisfaction (ratings of 4 or 5), dissatisfaction (ratings of 1 or 2), and neutrality (ratings of 3). Data are shown for all years, subjects, and institutions combined (total *n* = 2,741,654 responses). A few of the longer survey items were slightly abbreviated for the figure with no change to their meaning

The predictors of dissatisfaction and neutrality were identified by calculating the amounts of variation explained by survey items (Fig. 2). Explanation of variation was dominated by the organisation and management item ‘The course is well organised and running smoothly’ (Q15), tougher with items enquiring about perceptions of teaching, notably Q1 ‘Staff are good at explaining things’ and Q4 ‘The course is intellectually stimulating’. Ratings of courses running smoothly (Q15) were also the strongest predictor of neutrality and to a greater degree than with agreement and disagreement metrics. The teaching dimension was most strongly associated with dissatisfaction (i.e. when it was perceived as poor) together with low ratings of the support item Q11 (‘I have been able to contact staff when I needed to’) and the personal development item Q21 (‘As a result of my course I feel confident in tackling unfamiliar problems’). In the latter case, the perceived lack of confidence expressed in Q21 was more dissatisfying when rated low rather than its presence being perceived as satisfying. All other items made minimal contributions to the variation overall satisfaction and are considered to be little more than ‘noise’ in the model.

### Illustrative league table based on dissatisfaction

The survey instrument was not designed for comparing institutions wholesale or for its outputs being incorporated into calculations of simplistic league tables (Callender et al., 2014). There is a very strong quantitative rationale that institutional output metrics from the NSS should not be used for calculations that lead to rankings. There is greater variation within institutions than between them and a lack of statistical differences between institutional outcomes other than at the extremes of the dataset (Cheng & Marsh, 2010; Surridge, 2008). However, as Simpson (2018; p.1345) articulates well, “surveys beget rankings”, and survey ratings are routinely incorporated into third party, ranked ‘good university’ guides that have significant influence (Turnbull, 2018). The UK’s teaching excellence framework uses metrics from some of the NSS instruments as part of the calculation to simplistically rank institutions as bronze, silver, or gold. Therefore, as much as the authors agree that league tables and rankings can be misleading, we feel justified in providing a table of ranked institutions for illustrative purposes only. This hypothetical exercise is for research purposes and should be viewed only as a way to demonstrate change that results from shifting emphasis to metrics of disagreement.

Table 2 highlights ranking differentials of a selection of institutions after switching emphasis from measures of satisfaction to dissatisfaction. Change in rankings overall represents a zero-balance calculation, so the ‘winners and losers’ are balanced out in terms of net positional changes. In the extremes, there was minimal change in ranking of the very highest and lowest ranking institutions based on satisfaction when a lack of dissatisfaction became the premise. However, below these stable extreme groupings, institutions began to show marked changes, particularly ‘mid-ranked’ grouping (representing original rankings positioned 66–85; Table 1b), which contained the institution that would have most benefited for the whole dataset, Institution ‘AAAA’. This extremity climbed 47 places in the league table once the refocus was positioned on a lack of dissatisfaction. The third ranked institution originally (institution ‘J’) dropped seven places to 10th, which shows significant changes occurred in the highest ranked institutions. The greatest drop in ranking was an institution (not shown in the selected groupings) that would have dropped from a ranking of 22nd based on satisfaction to a ranking of 55th if an absence of dissatisfied participants was the focus.

**Table 2:**
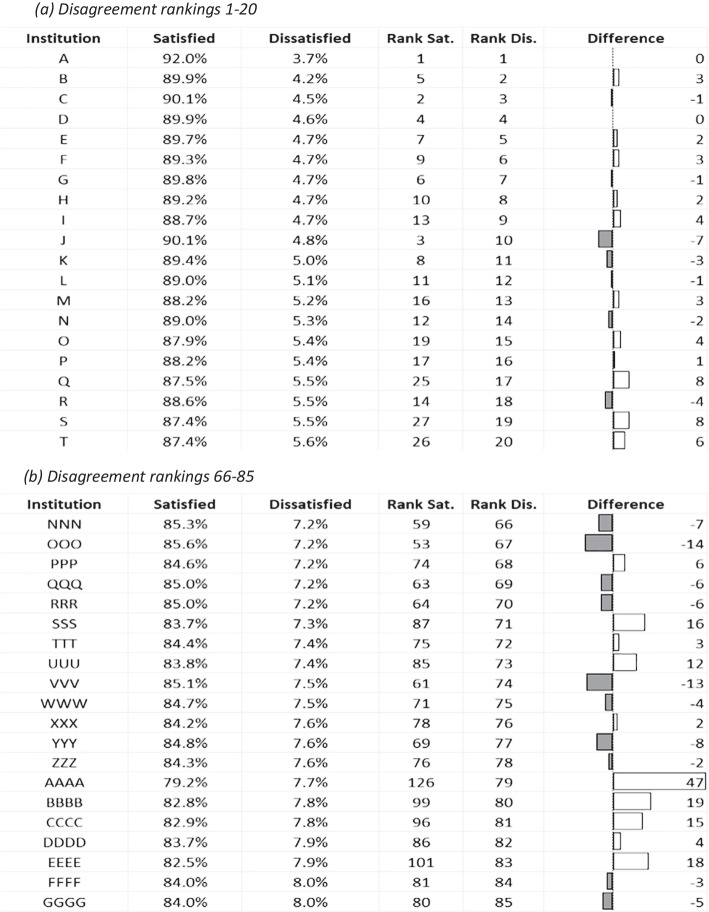

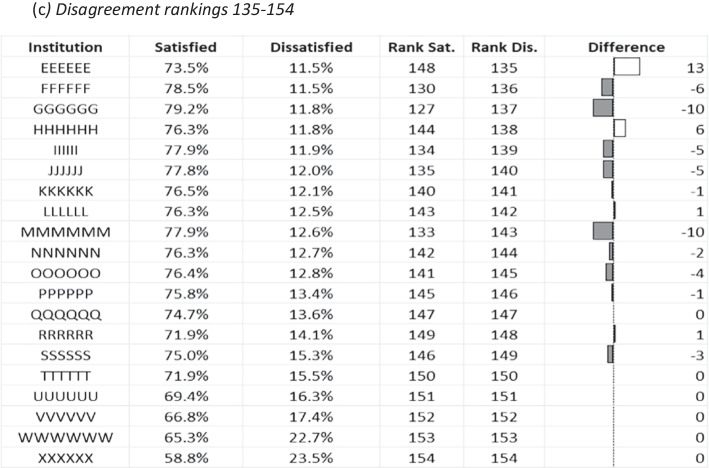
An illustrative table to show institutional rankings based on percentages of both overall satisfaction (most to least based on the single survey item enquiry directly about satisfaction with the course) and overall dissatisfaction (least to most) based on 12 years of outcomes from the original survey instrument. For illustrative purposes, a selection of the 20 highest and lowest ranking institutions, together with a middle grouping, is shown. Overall dissatisfaction outcomes are shown alongside their relative change in rankings (Rank Dis.), providing a relative difference that would result if the emphasis was moved away from the notion of satisfaction (Rank Sat.)

## Discussion

This longitudinal exploration of a national student survey has demonstrated several outcomes for the first time. Firstly, the determinants of dissatisfaction and neutrality metrics were, to a large degree, both stable over time and mimicked that of student satisfaction (cf. Langan & Harris, 2019). Secondly, the widely publicised year-on-year increases in student satisfaction metrics have been driven by complex reductions in neutrality and disagreement from across different survey dimensions. Finally, an illustrative league table developed from the longitudinal dataset has illustrated how a notional shift to the usage of dissatisfaction metrics would have impacted significantly on relative rankings of many UK universities.

### Predictors of dissatisfaction

There was a clear set of metric predictors of overall dissatisfaction that are similar, but not identical, to those known to underpin overall satisfaction metrics (Burgess et al., 2018; Langan & Harris, 2019). For the entire lifespan of the original instrument, variation in Q22 was dominated by items enquiring about perceptions of the course running smoothly and aspects of the taught experience (see Langan et al., 2013). The trend was apparent for all three of the broad metric outcomes: satisfaction, dissatisfaction, and neutrality, and the overarching influence of perceptions of course organisation is supported by explorations of respondent free text comments (Langan et al., 2017).

Aspects of teaching (the other main influential dimension of the survey instrument) and course organisation have been highlighted as influential in other types of student survey. Notably, the UK’s HEPI Student Academic Experience Surveys (https://www.hepi.ac.uk/) consider wider aspects of the HE experience. Several recent HEPI reports reported experiences of teaching as most influential of student attitudes about perceptions of ‘value for money’. These surveys also provide evidence that factors not measured in the NSS, such as teaching contact hours, the likelihood of getting a well-paid graduate job, and personal well-being, have greater impacts on the quality of the student experience than course organisation. This approach exposes limitations of relying on single survey instruments such as the NSS for decision-making, despite being the largest in the UK in terms of participation rates and most influential (Burgess et al., 2018).

We acknowledge several key constraints on our interpretations, including that survey instruments such as the NSS are highly constrained in the comprehensiveness of the information that they can harvest about the student experience and bespoke to the HE ecosystem they are exploring (Wilson et al., 1997). In addition, the current study provides a high-level synthesis, and there may be more subtle, granular effects such as differences between subjects or participant demographics (Burgess et al., 2018; Langan et al., 2013). In addition, we explored long-term data from the original UK survey instrument, and this instrument has now been modified. Satterthwaite and Roudsari (2020) explored the UK’s updated NSS instrument and found that, together with the perceived value of teaching quality, a new category of ‘learning opportunities’ was of significant importance. Parapadakis (2020) used related artificial intelligence approaches to explore NSS metrics from Computer Science courses and agreed generally with our predictors, whilst also highlighting differences in predictive factors between institutional typologies and institutional rankings.

The validity of using simple metrics to explore notions of (dis)satisfaction is, of course, disputable, but they provide insights into university students (Langan, 2022) and are relatable to other forms of research. Douglas et al., (2006, 2008, 2015) revealed perspectives of notions of student satisfiers and dissatisfiers using critical incident analyses, questionnaires, and hand-written narratives that are relatable with the outcomes of the current study. They positioned the student experience as a ‘service-product bundle’ that consists of elements such as physical or facilitating goods (including teaching activities and environments), explicit ‘sensual’ services (such as teaching quality), and implicit psychological services (such as staff friendliness). The most impactful themes identified in Douglas et al. (2006) were around the themes of teaching ability of staff, and, subsequently, in Douglas et al. (2015), the themes of communication (strongest dissatisfier) and attentiveness to student needs (strongest satisfier) were of highest importance. Many of the other aspects of the student experience were perceived as largely ‘neutral’ in the interpretation, relating to the ‘noise’ of many items in our models predicting (dis)satisfaction. We suggest that the key determinants in Douglas’ works can be linked to the main NSS dimensions identified as predictors in the current study: notably, teaching quality, student support, and, to a lesser extent, course management (Langan & Harris, 2019). Adopting this terminology in the current study, the most influential item pertaining to perceptions that students’ courses are running well was the only example of an item being more of a satisfier when it was perceived as happening than a dissatisfier (when perceived as lacking). Conversely, the teaching experience seems to have been more of a dissatisfier when perceived as poor quality than a satisfier when perceived as high quality. The nuanced outcome of some factors being more ‘dissatisfying than satisfying’ was also the case for items enquiring about advice and support, being able to contact staff and confidence in problem-solving. Douglas et al. (2015) suggest institutions should focus on satisfiers as a means to improve the service for their ‘primary customers’, whereas our outcomes suggest both dissatisfaction and satisfaction could be considered together as they represent different groups of participants with different viewpoints and needs.

### Drivers of metric gains in satisfaction

The well-publicised national increases in student satisfaction in the UK during the lifetime of the original survey instrument were driven by reductions in levels of both neutrality and disagreement. We suggest that this scenario is preferable to one where the increase in satisfaction was drawn from neutral changing to express agreement with unchanged levels of disagreement/dissatisfaction. Since the inception of the national survey, there has been a mantra in UKHE of ‘moving from threes to fours’ (Kernohan, 2019). This has been in reference to the survey’s Likert scale of 1 to 5, where 4 is the threshold to be considered an agreeable response. Thus, there was a possibility of reduction in neutrality but not dissatisfaction. Potential ‘ceilings’ to levels of satisfaction were identified by Langan and Harris (2019), and the inverse in the current study is ‘floors’ that are apparent in some dimensions based on disagreement/ dissatisfaction (notable in both teaching and personal development).

The flattening of outcomes occurs towards the end of the original survey instrument’s lifetime suggests a potential difficulty to reach population (somewhere in the region of about 5% of respondents for satisfaction) was prevailing before the survey instrument was reviewed and modified. This situation prompts questions of how to further reduce the levels of disagreement and whether institutions will focus on this group if disagreement metrics are not reported directly. The notion of a resilient dissatisfied group raises a possibility that student dissatisfaction may qualify as a wicked problem that will not be entirely solved (Rittel and Webber, 1973), a scenario that has been suggested for other HE challenges (e.g. student withdrawal; Hamshire et al., 2019). This research flags a need to explore the new survey instrument when it has been administered for sufficient time to explore longitudinal trends. There are already documented perturbations of the UK’s student views, including drops in metric outcomes of the updated NSS instrument and other national-level survey outcomes, during the global coronavirus pandemic captured that remain under scrutiny (Gibbons, 2022).

### Use of disagreement metrics

We suggest that there is need for debate about the public visibility of disagreement metrics if agreement metrics are to continue to be utilised and reported publicly. Disagreement and dissatisfaction provide a wider view of student survey outcomes (Cadotte & Turgeon, 1988) and, potentially, could enhance data-driven decision-making (see Hazelkorn, 2015 for challenges to this practice). It is acknowledged that there are risks associated with emphasising dissatisfaction, including fuelling discourse about students as ‘displeased customers’, at least in some contexts of their university experiences (Budd, 2017). This approach could exacerbate known issues with neoliberalism in the sector, such as the impacts of surveys on staff mental health (Thiel, 2019) and this in the context that students may not be fully aware of what is in their best interests (Spence, 2019). Dissatisfaction metrics could unfairly highlight the views of few individuals if they are the focus, be seen out of context, and disproportionally impact staff mental health meaning that student-customer satisfaction is not a worthy aim for colleges and universities (Arum & Roksa, 2014; p.2561).

The use of tabulation of rankings that we have provided is purely a mechanism to demonstrate how disagreement metrics could influence the UK HE sector outcomes, simply ranking them to show change. If dissatisfaction metrics were available, we suggest that it is extremely likely in the UK that some third-party providers would rank institutions in similar fashion to the use of agreement metrics. We do not support compounding these metrics into rankings (Callender et al., 2014) and acknowledge that the disagreement metrics represent only a small proportion of respondent views in the NSS (less than about 15% of the sample population). It is arguable that, despite being a small proportion of the sample population, these are important voices to be recognised, representing several hundreds of thousands of non-satisfied individuals over the survey’s lifetime. The hypothetical exercise showed how there would be noticeable changes in ranked outcomes, and the most likely to be affected were institutions in the ‘squeezed middle’ and not those in the extremes of the dataset. There is also consideration that the competition for improved league table positions means that institutions need to change views/experiences of neutral and dissatisfied individuals in what seems like a ‘hard-to-reach’ group, potentially creating the wicked problem of tackling student satisfaction, as there may be for student withdrawal (cf. Hamshire et al., 2019).

The current study prompts the question as to whether disagreement/dissatisfaction metrics should accompany metrics of agreement/satisfaction. Consumer ratings that are negative are highly influential even if they represent a tiny proportion of the response population, such as looking at the most negative reviews of products (Filieri et al., 2019). Outliers in the current study show that some institutions have a significant number of students who expressed non-satisfaction with their courses. It is debatable whether universities should place more value on the majority vote of satisfaction or the less-palatable minority who are dissatisfied. In one sense, we suggest that it is useful for stakeholders to have more information and greater context as long as they are contextualised in terms of the sample population they represent. On the other hand, authors such as Thiel (2019) and Sabri (2013) provide strong views that student surveys can have impactful negative impacts, notably on academic staff.

Tensions between the processes of marketisation of HE, financial drivers, access for greater numbers, diversity of students, and the (dis)satisfaction of all stakeholders are beyond the limits of the evidence base of the current study. We do, however, provide evidence of the changes in non-satisfied participants for the lifespan of a national survey instrument and suggest the outcome need context for meaningful interpretation. We also acknowledge that practices such as third-party rankings are an unsubstantiated reality of modern some HE cultures. Arguably, avoidance of reporting and utilising dissatisfaction metrics could be viewed in the same light as when HE practitioners make difficult decisions in the design of higher learning programmes that enhance learning gains but are not popular with students (e.g. Boehler et al., 2006).

There remains a need to clarify the effects at a more granular scale in terms of separating analyses of the ratings of disagreement (1 and 2) and agreement (4 and 5). Amongst other outcomes, this would highlight drivers of extreme dissatisfaction (aligned with the concept of ‘nay sayers’; see Blamey et al., 1999). It has been suggested that the anchors ‘definitely agree’ and ‘mostly agree’ are less of an issue and can be combined for reporting as a measure of satisfaction, whereas ‘definitely disagree’ and ‘mostly disagree’ are distinct and should not be combined as a measure of dissatisfaction (Higher Education Funding Council for England, 2014). This suggests more research is required to determine how dissatisfaction is usefully reported if it is agreed to be beneficial as a notional concept.

In the support of providing this type of information, it is obvious that some prospective students may wish to know the levels of dissatisfaction for particular courses. Views of displeased customers, in general, are highly valued (Gavilan et al., 2018), for example the negative impact of 1-star reviews has been shown to be greater than the positive impact of 5-star reviews (Papathanassis & Knolle, 2011). The availability of dissatisfaction data would provide a more comprehensive representation of student views than the focus on agreement. In terms of league table rankings, even with a wholesale move to disagreement from agreement, HEIs in the extremes of the rankings would likely see very little difference in this notional change. The highest-ranking institutions had little potential for change due to the low proportion of neutrality and disagreement. Despite potential ‘headspace’ for improvement in levels of agreement, the very lowest-performing institutions in this context were ‘significantly lower’ than counterparts that sat only slightly higher in the rankings and would thus need substantial change to enhance rankings (see Cheng & Marsh, 2010). It is important to stress again that, although the survey outcomes are not designed for rankings, they are most impactful when third parties do this (Hazelkorn, 2015). We suggest it is necessary to frame the point in this manner as a means to emphasise the potential of a change of ethos.

There is a hypothetical argument that students that have made the decision to express disagreement would be more difficult to influence unduly than their ‘neutral’ counterparts as a means to enhance the survey’s outcomes. There is evidence that institutions have used techniques to inflate the survey outcomes, albeit much of this is in mainstream media (Newman, 2008). These include signals to students to consider “what do you have to lose” by returning high metrics, whereas poor NSS outcomes may impact the reputation of their home institution and, consequently, negatively affect their own life chances (Adisa et al., 2022). There are many opportunities and means to overtly or covertly influence respondents to be more positive (see guidance for avoiding undue influence by Ipsos MORI, 2019). There also remains a possibility that Campbell’s Law is in effect, namely that ‘the more any quantitative social indicator is used for social decision-making, the more subject it will be to corruption pressures and the more apt it will be to distort and corrupt the social processes it is intended to monitor’ (Sidorkin, 2016).

## Conclusions

This study has confirmed that a narrow range of aspects of the student experience captured by a national level survey drove dissatisfaction metrics. The most influential items were related to course management and teaching quality, a finding that is consistent with predictors of satisfaction metrics. Most items were more dissatisfying when perceived as poor or absent than satisfying when effective or present. This was apart from the most influential factor of a course running smoothly, which was more of a satisfier when considered to have occurred. Diminishing levels of reported neutrality and disagreement that drive agreement metric enhancements were dependant on the survey dimension, led to the increasing levels of positive responses nationally. Outliers show that some institutions are under and overperforming in the UK sector in terms of levels of dissatisfaction, representing many students who have invested in the experience. It is acknowledged that our findings are derived from the original UK NSS survey instrument (see Richardson, 2005), and we cannot account for more recent turbulence in the outcomes of the new survey instrument or impacts on outcomes of the new survey dimensions (see Pollett & Shepherd, 2022). We suggest that it is worth the risk here of perpetrating neoliberal ideologies by providing our anonymised table of institutional disagreement rankings. However, this is just to *raise the question* and generate debate about whether the negative voices that emanate from a small proportion of the survey outcomes should be captured and considered alongside agreement metrics.
